# MicroRNA-585 inhibits human glioma cell proliferation by directly targeting MDM2

**DOI:** 10.1186/s12935-020-01528-w

**Published:** 2020-09-29

**Authors:** Wangsheng Chen, Lan Hong, Changlong Hou, Yibin Wang, Fei Wang, Jianhua Zhang

**Affiliations:** 1 Department of Radiology, Hainan General Hospital/Hainan Hospital of Hainan Medical University, Haikou, 570311 China; 2 Department of Gynecology, Hainan General Hospital/Hainan Hospital of Hainan Medical University, Haikou, 570311 China; 3 Department of Radiology, Shanghai East Hospital, Tongji University School of Medicine, No 150, JiMo Road, Pudong New Area, Shanghai, 200120 China

**Keywords:** MicroRNA-585, Human glioma cell, Proliferation, MDM2, MRI

## Abstract

**Background:**

MicroRNAs (miRNAs) are important regulators for cancer cell proliferation. miR-585 has been shown to inhibit the proliferation of several types of cancer, however, little is known about its role in human glioma cells.

**Methods:**

miR-585 levels in human glioma clinical samples and cell lines were examined by quantitative real-time PCR (qRT-PCR) analysis. Cell proliferation was measured by Cell Counting Kit-8 (CCK-8) and EdU incorporation assays in vitro. For in vivo investigations, U251 cells were intracranially inoculated in BALB/c nude mice and xenografted tumors were visualized by magnetic resonance imaging (MRI).

**Results:**

miR-585 expression is downregulated in human glioma tissues and cell lines compared with non-cancerous counterparts. Additionally, miR-585 overexpression inhibits and its knockdown promotes human glioma cell proliferation in vitro. Moreover, miR-585 overexpression also inhibits the growth of glioma xenografts in vivo, suggesting that miR-585 may act as a tumor suppressor to inhibit the proliferation of human glioma. Furthermore, miR-585 directly targets and decreases the expression of oncoprotein murine double minute 2 (MDM2). More importantly, the restoration of MDM2 via enforced overexpression markedly rescues miR-585 inhibitory effect on human glioma cell proliferation, thus demonstrating that targeting MDM2 is a critical mechanism by which miR-585 inhibits human glioma cell proliferation.

**Conclusions:**

Our study unveils the anti-proliferative role of miR-585 in human glioma cells, and also implicates its potential application in clinical therapy.

## Background

Malignant gliomas are the most common type of brain tumors characterized by aggressive progression and chemoresistance, causing extremely poor clinical prognosis and high mortality [1–3]. Although rapid progress has been obtained in diagnostic methods and therapies in recent decades, the prognosis of malignant gliomas has not been significantly improved, and many patients succumb to the disease within 5 years after diagnosis [4, 5]. To identify novel therapeutic targets and develop more effective strategies for treating malignant gliomas, it is imperative to gain a better understanding of the molecular mechanisms that underlie glioma progression.

Through regulating the expression of target genes, microRNAs (miRNAs), a class of small non-coding RNAs, play important roles in various cellular processes, such as proliferation, differentiation, apoptosis and migration [6]. Recently, accumulating evidence has shown that the deregulation of several miRNAs is involved in the tumorigenesis and development of a broad range of cancers [7], including gliomas [8, 9]. For instance, miRNA-184 inhibits glioma cell proliferation and invasion [10], and miRNA-370-3p induces glioma cell cycle arrest [11]. In addition, miR-136 promotes glioma cell apoptosis [12], and miR-141-3p promotes glioma cell temozolomide resistance [13]. Furthermore, miR-153-3p was demonstrated to enhance glioma cell radiosensitivity [14]. These findings suggest that miRNAs are potential therapeutic targets for glioma treatment.

Lately, some studies have reported that miR-585 acts as a tumor suppressor in non-small-cell lung cancer (NSCLC) [15] and gastric cancer [16]. However, in myeloma, miR-585 inhibition reduced tumor size in an animal model [17], hence suggesting complicated activities miR-585 may exert in different cancer types. Yet, as far as we know, the role of miR-585 in human glioma cells has not been explored. In this study, we report an anti-proliferative function of miR-585 in human glioma cells, where the targeted MDM2 constitutes a critical mechanism.

## Materials and methods

### Cell lines and human glioma tissues

The human U251, A172, LN229 and TJ899 glioblastoma cell lines and normal human glial cell line HEB were purchased from the American Type Culture Collection (ATCC) (Manassas, VA, USA). All cell lines were cultured at 37 °C in the Dulbecco’s modified Eagle’s medium (DMEM) (Invitrogen) supplemented with 10% fetal bovine serum (FBS) (Hyclone) in a humidified atmosphere of 5% CO_2_. Fifteen low-grade and eighteen high-grade glioma patients were recruited from Shanghai East Hospital, Tongji University School of Medicine. The written consent was obtained from each patient. The glioma tissues and surrounding normal brain tissues were separated under a microscopy, and confirmed by hematoxylineosin (HE) staining. The study was conducted according to the Declaration of Helsinki. The protocols were approved by the Ethics Review Board of Shanghai East Hospital, Tongji University School of Medicine.

### Quantitative real-time PCR analysis

The total RNA was extracted from normal brain tissues, glioma tissues, and glioma cell lines with the Trizol reagent (ThermoFisher Scientific). For quantifying miR-585 level using the quantitative rea-time reverse transcription PCR (qRT-PCR), RNA was transcribed into complementary DNA and amplified with specific sense primer 5′-ACGCGTTCTCCTTACCATCCCTGA-3′ and antisense primer 5′-CGATCTGGAAGTAACCCAAGCC-3′, based on a two-step qRT-PCR using the miRNA PrimeScript RT Master Mix kit (Takara) and SYBR Premix Ex Taq II Kit (Takara) according to the manufacturer’s instructions. U6 snRNA was used as an internal control. Each PCR reaction included three replicates and three independent experiments were performed.

### Lentiviral-mediated expression and knockdown

The lentiviral vector expressing scrambled RNA (Lev-control), expressing miR-585 (Lev-miR-585) and expressing shRNA targeting miR-585 (Lev-shmiR-585) were purchased from GeneChem (Shanghai, China). A172 and U251 cells were infected with Lev-control, Lev-miR-585 or Lev-shmiR-585, and stably infected cells expressing green fluorescent protein (GFP) were selected by the fluorescence-activated cell sorting flow cytometry (FACSAria II, BD), and expanded in culture for further experiments. The total RNA was isolated from cell clones, and miR-585 expression was determined using RT-qPCR analysis to verify the efficiency of miR-585 overexpression and knockdown.

### Cell proliferation measurement

Cell proliferation was measured by CCK-8 assay and EdU incorporation assay. For CCK-8 assay, A172 and U251 cells were seeded in 96-well plates, and cell viability was eveluated using the Cell Counting Kit-8 kit (C0038, Beyotime) according to the manufacturer’s instructions. Cell viability was expressed as the percentage of control group. The EdU incorporation assay was conducted as previously reported [18]. In brief, proliferating A172 and U251 cells cultured in 24-well plates were examined using the Click-iT Edu Proliferation Assay Kit (C10337, ThermoFisher Scientific) according to the manufacturer’s instructions. The Edu incorporation was analyzed by the flow cytometry (Accuri C6, BD). Each group included five replicates and three independent experiments were performed.

### Western blot analysis

Western blot analysis was performed as described previously [19]. Briefly, in each lane, 50 µg proteins were loaded and separated by SDS-PAGE, and then transferred on PVDF membranes were probed with MDM2 antibody (sc-965, Santa Cruz) or β-Actin antibody (sc-47777, Santa Cruz), which were further probed with the HRP-conjugated goat anti-mouse IgG secondary antibody (sc-2005, Santa Cruz). The protein blots were developed using the enhanced ECL chemiluminescence reagents (32106, Pierce).

### Tumor xenograft model

U251 cells (5 × 10^5^) stably infected with Lev-control or Lev-miR-585 were intracranially inoculated in male BALB/c nude mice. Seven mice were included in each group. The mice were maintained in a pathogen-free facility throughout this experiment. At 2 weeks after inoculation, tumors were visualized by magnetic resonance imaging (MRI) technique and T_2_-weighted imaging (T2WI) was obtained as previously described [20]. All animal procedures were performed in accordance with the protocols approved by the National Institutes of Health Guide for the Care and Use of Animals.

### Immunohistochemistry

The paraffin sections with 4-mm thickness were prepared from xenografted tumors. Before antibody staining, sections were deparaffinized in pure xylene, rehydrated in serial ethanol and water, underwent heat-induced antigen retrieval in 10 mM citrate buffer for 5 min, and blocked with 2% hydrogen peroxide and 5% goat serum. Sections were incubated overnight with Ki-67 antibody (27309-1-AP, Proteintech) or MDM2 antibody (SAB4300601, Sigma-Aldrich) at 4 °C. After washing with PBS, sections were incubated with biotinylated goat anti-rabbit IgG antibody (ab64256, Abcam) for 1 h at room temperature, and further incubated with HRP streptavidin (RABHRP3, Millipore). Sections were incubated with 3,3-diaminobenzidine (DAB) to trigger peroxidase reaction, then counterstained with hematoxylin and mounted in neutral gum. The stained sections were analyzed using a bright field microscope.

### Luciferase reporter assay

The 3′-UTR fragment of human MDM2 gene with the predicted miR-585 binding sites was amplified by PCR and cloned into pmIR-ReporterTM firefly luciferase reporter vector (Genepharm), which was used as a wide-type construct (WT). The site-directed mutation of miRNA-585 binding sites within the MDM2 3′-UTR was performed with GeneTailor Site-Directed Mutagenesis System (Invitrogen) to develop the mutant construct (Mut). For each well, 100 ng WT or Mut construct was cotransfected into 5 × 10^4^ HEK293 cells with 20 nM miRNA-585 mimics or inhibitors, or with 20 nM siRNA negative control or siRNA miR-585, and the luciferase activity was measured at 48 h after transfection with the Dual-Luciferase Reporter Assay System (Promega) according to the manufacturer’s instructions. The activity of Firefly luciferase was used for normalization.

### Statistics

Data are expressed as the mean ± standard deviation (SD). One-way ANOVA followed by post hoc Bonferroni test or two-tailed unpaired Student’s t test (SPSS 18.0, SPSS Inc., Chicago, IL, USA) was used for comparisons between different groups. Differences with P < 0.05 were considered to be statistically significant.

## Results

### miR-585 expression is downregulated in human glioma tissues and cell lines

**Fig. 1:**
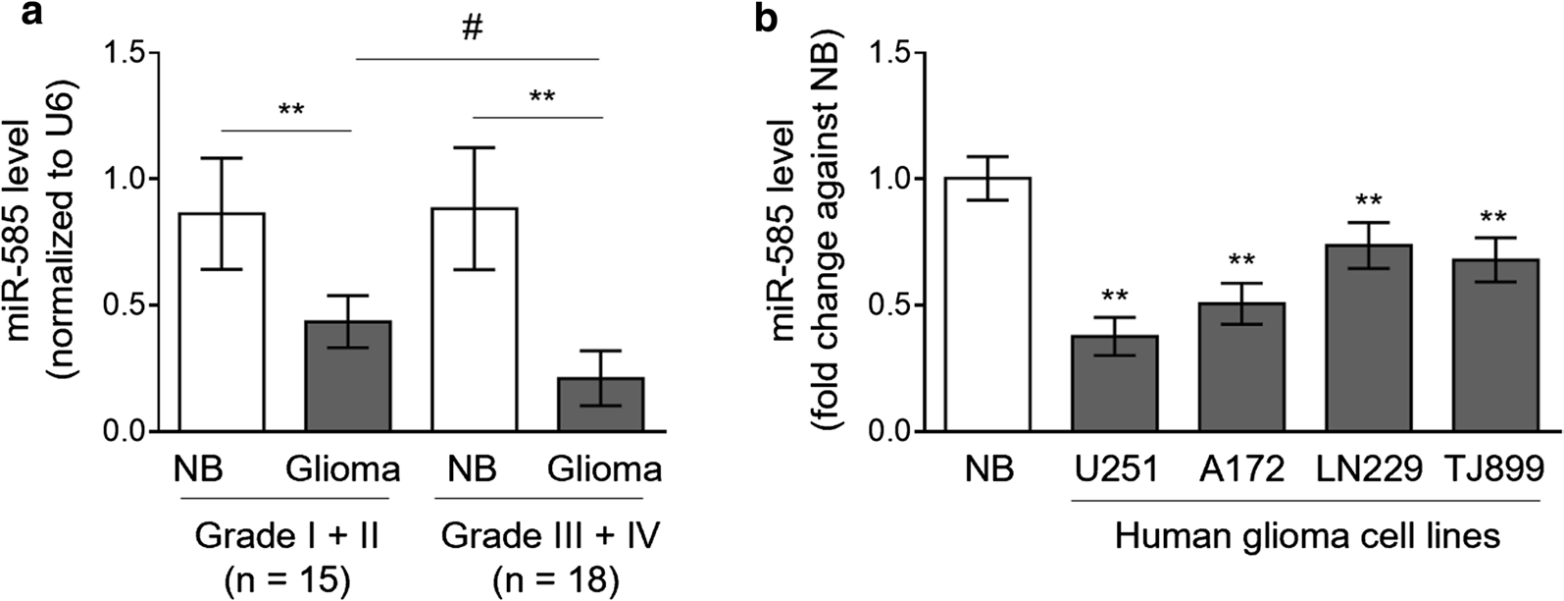
miR-585 is downregulated in human glioma tissues and cell lines. **a** qRT-PCR analysis of miR-585 expression in human glioma tissues (grade I + II, n = 15; grade III + IV, n = 18) and paired surrounding normal brain tissues (NB). Results are normalized to that of U6 snRNA internal control. Data are mean ± SD. Statistical analysis was performed by two-tailed Student’s *t* test for paired (**P < 0.01) and unpaired (^#^P < 0.01) data. **b** qRT-PCR analysis of miR-585 expression in 4 different human glioma cell lines as indicated. The normal brain (NB) total RNA was used as a control. Results are normalized to that of U6 snRNA and expressed as a fold change against NB. Data are mean ± SD (n = 3). Two-tailed unpaired Student’s *t*-test. **P < 0.01 (vs. NB)

To seek whether miR-585 is functionally involved in glioma pathogenesis, we firstly compared its expression levels between surrounding normal brain tissues and paired human glioma tissues by the quantitative real-time PCR (qRT-PCR) analysis. The human glioma tissues were classified by low-grade (WHO I–II; n = 15) and high-grade (WHO III–IV; n = 18) tumors [21]. As a result, compared with normal counterparts, a significant downregulation of miR-585 was observed in glioma tissues (*P* < 0.01), and this trend was even more significant in high-grade glioma tissues than low-grade ones (*P* < 0.05) (Fig. 1a, Additional file 1). To further relate miR-585 to glioma pathogenesis, its expression in a normal human glial cell line HEB was compared with four human glioma cell lines, including U251, A172, LN229 and TJ899. Similarly, we found that in contrast to HEB cells, the expression level of miR-585 was also decreased in all of these human glioma cell lines (Fig. 1b). Altogether, these results indicate that miR-585 is downregulated in both human glioma tissues and glioma cell lines, pointing to a potential functional role of miR-585 associated with human glioma.

### miR-585 inhibits glioma cell proliferation in vitro

**Fig. 2:**
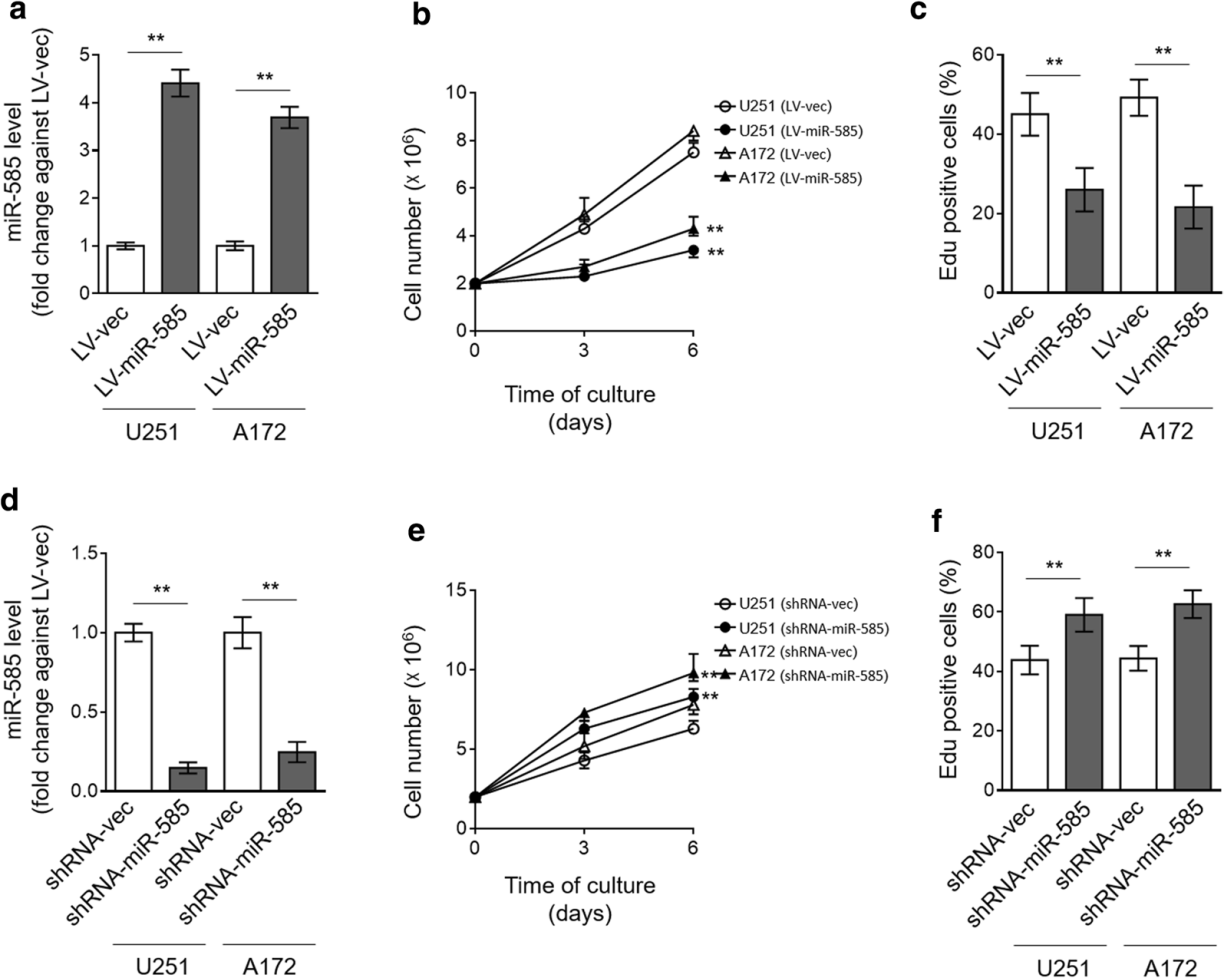
miR-585 inhibits proliferation of human glioma cells in vitro. **a**–**c** U251 and A172 cells were stably infected with lentivirus expressing empty vector (LV-vec) or miR-585 (LV-miR-585). **a** miR-585 expression was determined by qRT-PCR analysis. U6 snRNA was used as an internal control. Data are mean ± SD (n = 3). Two-tailed unpaired Student’s *t*-test. **P < 0.01. **b** The cell number of U251 and A172 cells cultured for consecutive 3 and 6 days was counted by CCK-8 assay. Data are mean ± SD (n = 5). One-way ANOVA followed by post hoc Bonferroni test. **P < 0.01 (vs. LV-vec). **c** The proliferating U251 and A172 cells were stained with EdU, and the percentage of EdU positive cells was analyzed by FACS assay. Data are mean ± SD (n = 5). Two-tailed unpaired Student’s *t*-test. **P < 0.01. **d**–**f** U251 and A172 cells were stably infected with shRNA-vec or shRNA-miR-585. The expression of miR-585 (**d**), cell number of U251 cells as assayed by CCK-8 (**e**) and EdU incorporation (**f**) were analyzed as same as in (**a**–**c**)

miR-585 has been shown to suppress the cell proliferation of NSCLC and gastric cancer [15, 16]. We wondered whether miR-585 imposes a similar activity on glioma cells. To test this possibility, miR-585 was overexpressed in U251 and A172 cells via lentiviral infection (Fig. 2a), and cell proliferation was then assessed by cell count kit-8 (CCK-8) assay. As shown, contrary to vector, U251 and A172 cells overexpressing miR-585 proliferated markedly slower (Fig. 2b). Likewise, EdU incorporation assay also showed that the proliferation of U251 and A172 cells was significantly suppressed upon miR-585 overexpression (Fig. 2c), together describing an anti-proliferative effect of miR-585 on glioma cells. In order to validate miR-585 function, we applied a technique of shRNA-mediated knockdown to deplete miR-585 expression in U251 and A172 cells (Fig. 2d). We found that consistent with the results obtained through miR-585 overexpression, oppositely, its depletion increased the proliferation rate of U251 and A172 cells, as revealed by CCK-8 (Fig. 2e) and EdU incorporation (Fig. 2f) assays. Hence, we conclude that miR-585 inhibits glioma cell proliferation, at least in vitro.

### miR-585 inhibits glioma cell growth in vivo

**Fig. 3:**
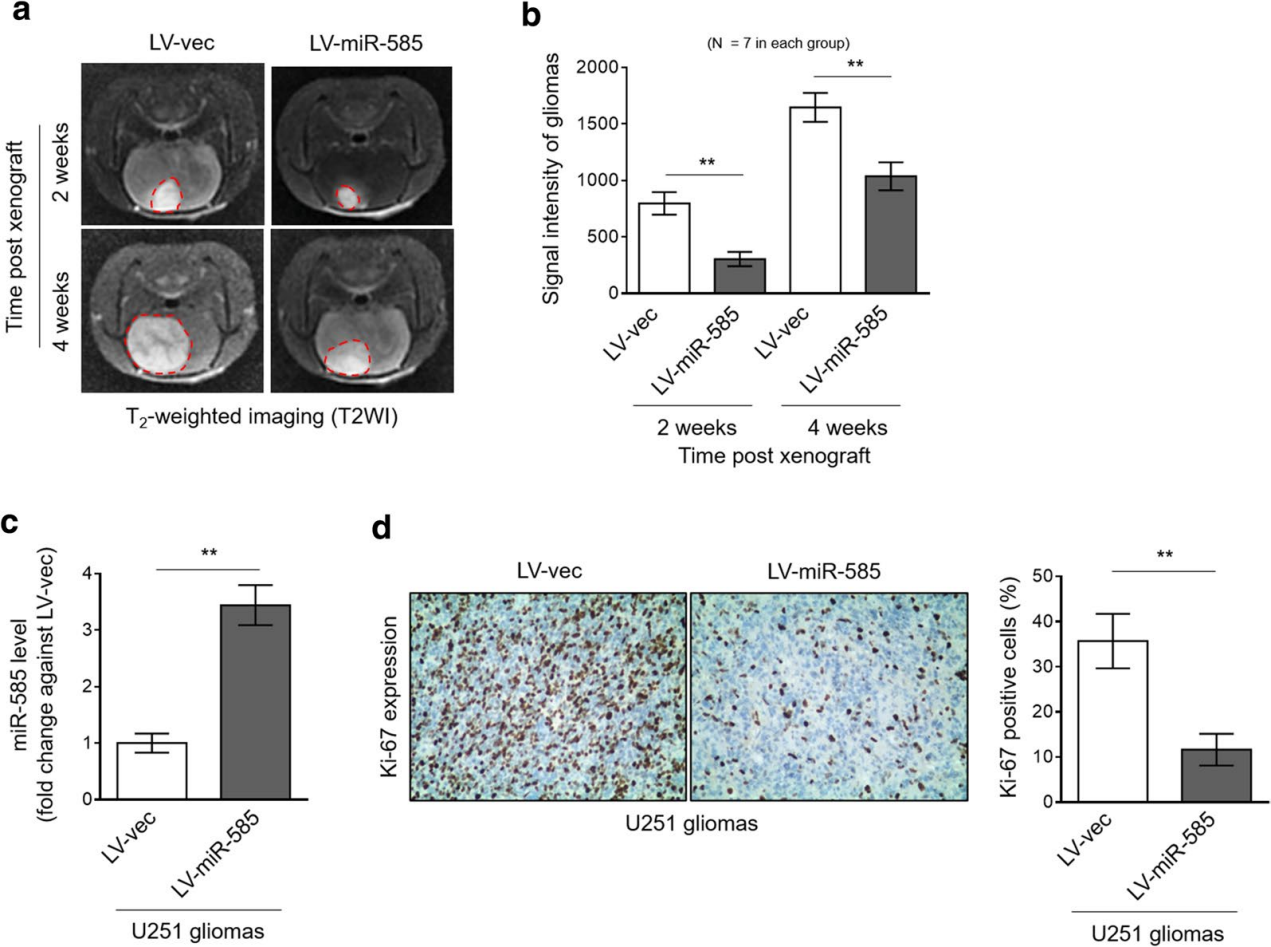
miR-585 inhibits growth of human glioma cells in vivo. **a–d** U251 cells stably infected with LV-vec or LV-miR-585 were intracranially inoculated in athymic nude (nu/nu) mice (n = 7 mice per group). **a** Representative T_2_-weighted imaging (T_2_WI) of mice with xenografted glioma after 2 weeks of inoculation was captured using 3.0 T MRI. **b** The signal intensity of T_2_WI as shown in (**a**) in each group was analyzed. Data are mean ± SD (n = 7). Two-tailed unpaired Student’s *t*-test. **P < 0.01. **c** miR-585 expression in isolated tumors was determined by qRT-PCR analysis. U6 snRNA was used as an internal control. Data are mean ± SD (n = 3). Two-tailed unpaired Student’s *t*-test. **P < 0.01. **d** Ki-67 expression in U251 tumor sections was analyzed by immunohistochemical (IHC) staining. The representative images (left) and percentage of Ki-67 positive cells (right) are shown. Twelve random fields from 3 sections of each group were quantified. Data are mean ± SD (n = 3). Two-tailed unpaired Student’s *t*-test. **P < 0.01

To examine whether miR-585 inhibits glioma cell growth in vivo, we xenografted U251 cells with or without miR-585 overexpression into BALB/c nude mice intracranially. As shown by the T_2_-weighted imaging (T2WI) obtained with 3.0 T magnetic resonance imaging (MRI) technique, in comparison to vector control, miR-585-overexpressing U251 tumors manifested lower growth rate (Fig. 3a, b). The overexpression of miR-585 in xenografted tumors was confirmed by qRT-PCR analysis (Fig. 3c). Moreover, consistently, immunohistochemistry analysis showed that the expression of Ki-67, a proliferation marker [22], was sharply decreased in xenografted U251 tumors along with miR-585 overexpression (Fig. 3d). Collectively, these data support a notion that miR-585 inhibits glioma cell growth in vivo.

### miR-585 directly targets MDM2

**Fig. 4:**
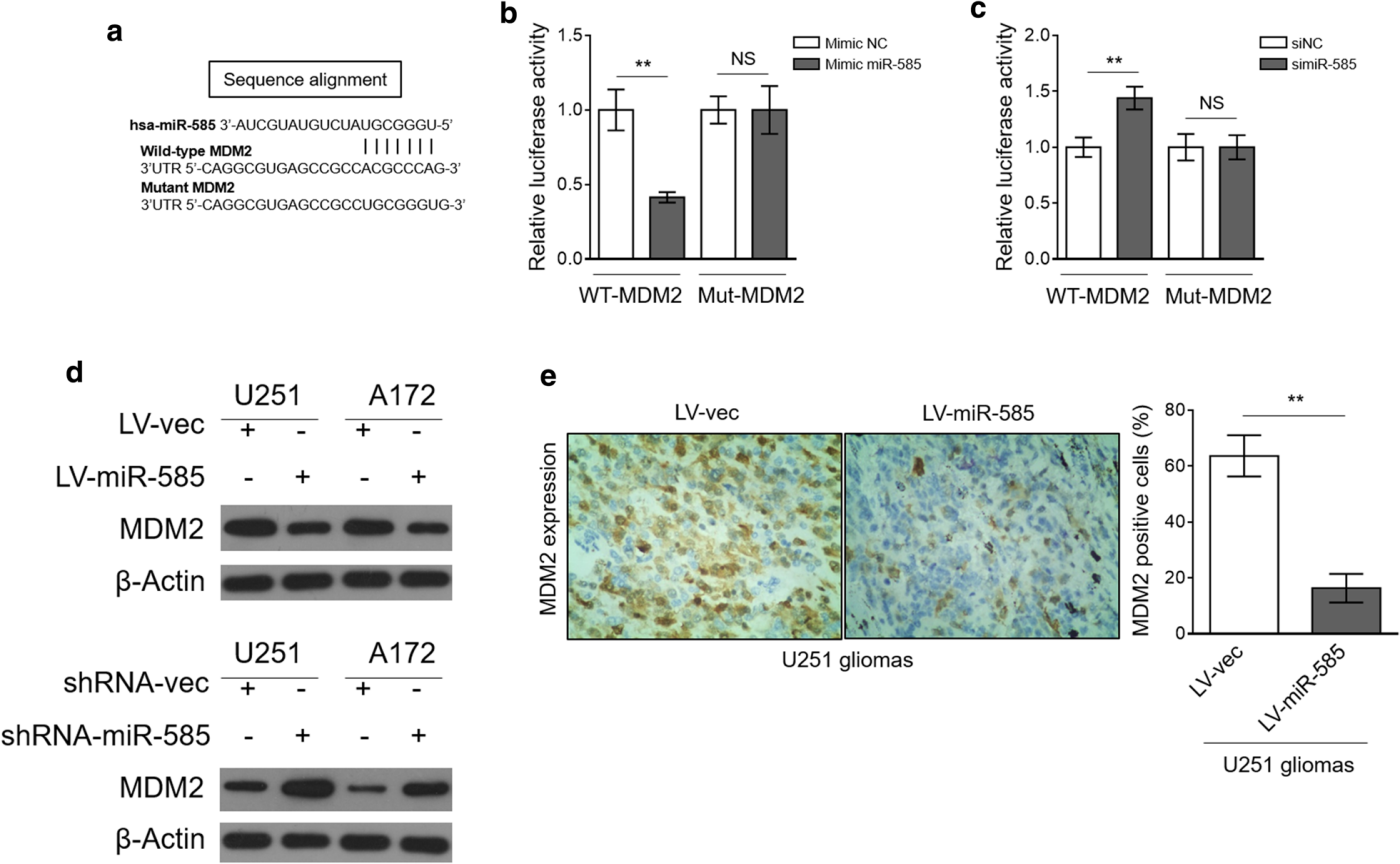
miR-585 directly targets MDM2. **a** The putative binding site of miR-585 in 3′-UTR of MDM2. **b** HEK293 cells were cotransfected mimic negative control (NC) or mimic miR-585 with firefly luciferase reporter containing wild type (WT) or mutant (Mut) 3′-UTR of MDM2. The luciferase activity was determined by dual-luciferase reporter assay. Data are mean ± SD (n = 3). Two-tailed unpaired Student’s *t*-test. **P < 0.01. **c** HEK293 cells were cotransfected siRNA negative control (siNC) or siRNA miR-585 with firefly luciferase reporter containing WT or Mut 3′-UTR of MDM2. The luciferase activity was determined by dual-luciferase reporter assay. Data are mean ± SD (n = 3). Two-tailed unpaired Student’s *t*-test. **P < 0.01. **d** The protein expression of MDM2 in U251 and A172 cells with miR-585 overexpression (upper) or knockdown (lower) was determined by Western blot analysis. β-Actin was used as a loading control. The representative images from 3 independent experiments are shown. **e** MDM2 expression in U251 tumor sections from Fig. 3d was analyzed by IHC staining. The representative images (left) and quantification analysis of MDM2 expression (right) are shown. Twelve random fields from 3 sections of each group were quantified. Data are mean ± SD (n = 3). Two-tailed unpaired Student’s *t*-test. **P < 0.01

MiRNAs function through base-pairing with the complementary sequences of target mRNAs to regulate gene expression at a post-transcriptional level [23]. To understand the molecular mechanism by which miR-585 inhibits glioma cell proliferation, we predicted its potential targets via the TargetScan algorithms [24]. Among the predicted targets, the murine double minute 2 (MDM2) is of great interest due to its important role in glioma cell proliferation (Fig. 4a) [25, 26]. Next, luciferase reporter assay was performed to test whether MDM2 is a direct target of miR-585. The results showed that miR-585 overexpression suppressed (Fig. 4b) and miR-585 knockdown elevated (Fig. 4c) the luciferase activity of wild-type MDM2 construct but not a mutant one, proving that miR-585 indeed directly targets MDM2. In accordance with this, miR-585 overexpression decreased MDM2 expression in glioma cells (Fig. 4d, upper), and reversely, its knockdown led to an increased expression of MDM2 (Fig. 4d, lower). Furthermore, remarkably, immunohistochemistry analysis revealed that MDM2 expression was downregulated in miR-585-overexpressing U251 xenografted tumors (Fig. 4e). These several lines of evidence demonstrate that miR-585 suppresses MDM2 expression in glioma cells through direct targeting.

### miR-585 inhibits human glioma cell proliferation by suppressing MDM2 expression

**Fig. 5:**
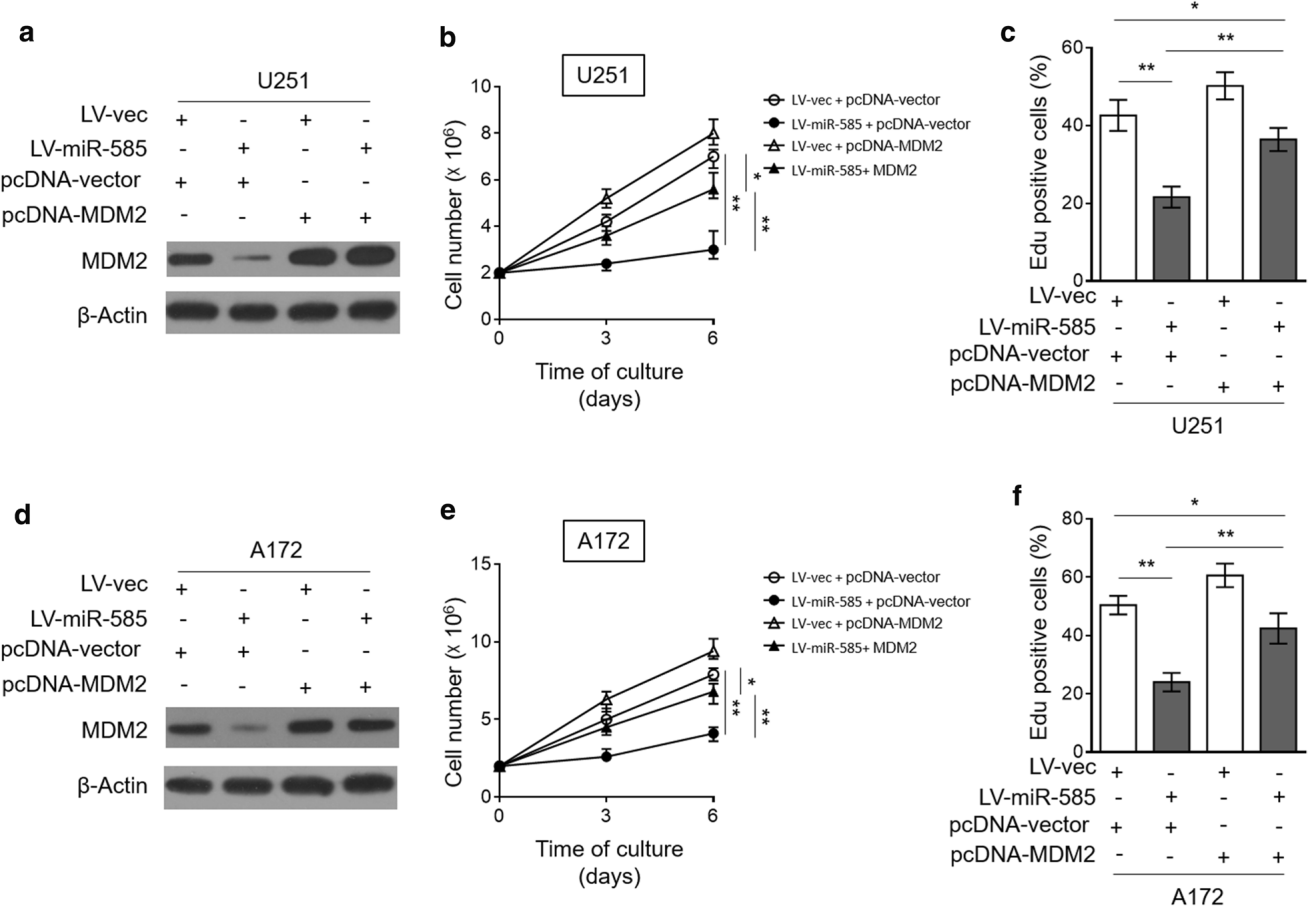
MDM2 restoration rescues inhibitory effect of miR-585 on human glioma cell proliferation. **a**–**c** U251 cells stably infected with LV-vec or LV-miR-585 were transfected with pcDNA3.1 vector or pcDNA3.1 MDM2. **a** The protein expression of MDM2 was determined by Western blot analysis. β-Actin was used as a loading control. The representative images from 3 independent experiments are shown. **b** The cell number of U251 cells cultured for consecutive 3 and 6 days was counted by CCK-8 assay. Data are mean ± SD (n = 5). One-way ANOVA followed by post hoc Bonferroni test. **P < 0.01; *P < 0.05. **c** The proliferating U251 cells were stained with EdU. The percentage of EdU positive cells was analyzed by FACS assay. Data are mean ± SD (n = 5). Two-tailed unpaired Student’s *t*-test. **P < 0.01; *P < 0.05. **d–f** A172 cells stably infected with LV-vec or LV-miR-585 were transfected with pcDNA3.1 vector or pcDNA3.1 MDM2. The protein expression of MDM2 (**d**), cell number of A172 cells as assayed by CCK-8 (**e**) and EdU incorporation (**f**) were analyzed as in (**a**–**c**)

To further shed light on the contribution of regulated MDM2 to miR-585 inhibitory effect on glioma cell proliferation, we restored MDM2 expression in miR-585-overexpressing U251 cells by transient transfection (Fig. 5a). CCK-8 assay showed that miR-585-mediated inhibition of cell proliferation was largely recovered in the presence of MDM2 restoration (Fig. 5b). Additionally, EdU incorporation assay also confirmed that MDM2 restoration diminished, although not completely, the anti-proliferative effect of miR-585 on U251 cells (Fig. 5c, Additional file 2). Furthermore, to collaborate these findings, we attempted to reproduce these phenotypes using A172 cells. Consequently, similar results were obtained when A172 cells were investigated (Fig. 5d–f, Additional file 2). Taken together, these mechanistic studies demonstrate that MDM2 is a critical target through which miR-585 inhibits glioma cell proliferation.

## Discussion

**Fig. 6:**
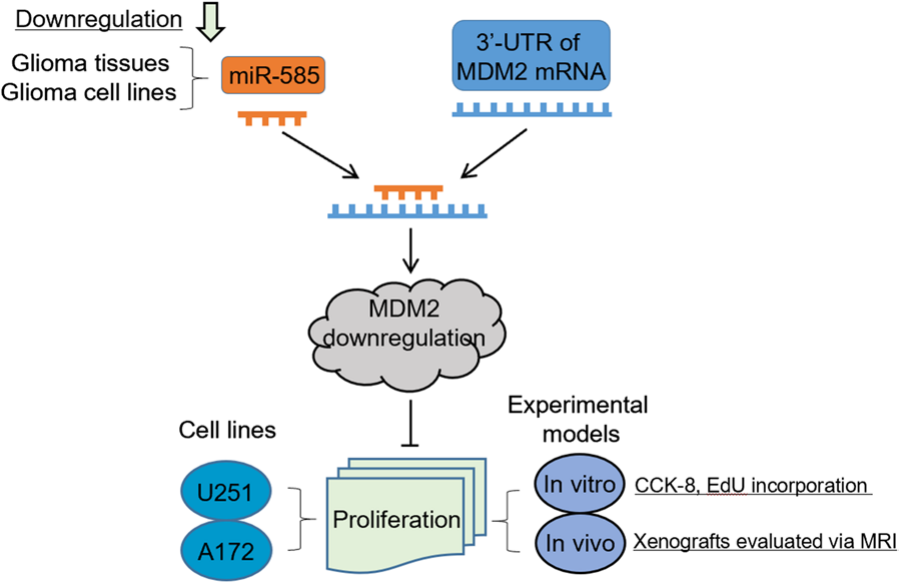
A proposed schematic model of study. miR-585, a downregulated miRNA in glioma tissues and cell lines, suppresses MDM2 level by directly binding to the 3′UTR of MDM2, leading to proliferation inhibition in glioma cell lines (U251 and A172) under both in vitro and in vivo experimental settings

The aberrant expression profile of miRNAs frequently appears in gliomas, and the deregulation of some miRNAs has been mechanistically linked with a variety of processes of glioma pathogenesis, including initiation, survival, proliferation, invasion, angiogenesis and apoptosis [27–29]. Yet, potential miRNAs involved in glioma pathobiology are not fully uncovered and many molecular mechanisms by which miRNAs contribute to gliogenesis remain to be elucidated. The expression and functional characteristics of miR-585 have been reported in oral cancer, gastric cancer and NSCLC [15, 16, 30]. In this study, we show a downregulated expression of miR-585 in human glioma tissues and glioma cell lines, and functionally, miR-585 inhibits glioma cell proliferation under in vitro and in vivo experimental settings. We further demonstrate that MDM2 is a direct target of miR-585 in glioma cells, and that the restoration of MDM2 substantially rescues miR-585 effect on glioma cell proliferation, thus establishing that the inhibitory effect of miR-585 on glioma cell proliferation depends on directly targeting MDM2 (Fig. 6).

To our knowledge, this is the first time the downregulation of miR-585 is reported in human gliomas. Coincidely, in a previous study, the decreased expression of miR-585 was found in primary NSCLC tumor tissues and cell lines [15]. Besides, similar results were reported in gastric cancer tissues and cell lines [16]. These observations together with our findings led us to suppose that miR-585 may serve as a tumor suppressor in a broad range of human cancer types. Nonetheless, it should be noted that the size of clinical samples and cell lines is relatively limited in the present study, future efforts with larger sample size would be necessary to consolidate miR-585 expression alteration in human gliomas. The dysregulated miRNAs in gliomas could be exploited as prognostic biomarkers, such as miR-182 [31], miR-326 [32] and miR-210 [33]. According to our observations, miR-585 downregulation is more significant in high-grade gliomas than low-grade ones, which suggests its expression is negatively correlated with the pathology classification of gliomas, it is thus of clinical significance to examine whether miR-585 expression is a possible prognostic marker for glioma patients. The changes in miRNA expression in cancer cells are presumably due to mechanisms occurring at a transcriptional level, including changes in gene expression and promoter hypermethylation, as well as changes in miRNA processing which belong to post-transcriptional mechanisms [34]. Interestingly, in oral squamous cell carcinoma (OSCC), miR-585 expression was found frequently silenced through a mechanism of tumor-specific DNA hypermethylation [30]. Therefore, we guess that the promoter hypermethylation of miR-585 gene is a possible mechanism that is responsible for its downregulation in glioma tissues and cells lines. Further studies are required to prove this conjecture.

miR-585 functions as a tumor suppressor in gastric cancer and NSCLC, as manifested by the inhibited proliferation, migration, and invasion of cancer cells when miR-585 is overexpressed, in which MAPK1 and hSMG-1 are molecular targets and mediate the tumor-suppressive effects of miR-585, respectively [15, 16]. We demonstrate that miR-585 inhibits glioma cell proliferation predominantly by suppressing MDM2 expression. These possibly describe that miR-585 exerts its effects through distinct targets in the context of different types of cancer cells. MDM2 is an oncoprotein that binds to the p53 tumor suppressor gene product and promotes its degradation [35]. The amplification and overexpression of MDM2 are present in human malignant gliomas and cell lines, independent of p53 status [36, 37], and high MDM2 expression is correlated with poor prognosis of glioma patients [38]. It has also been shown that miR-610 [25] and long non-coding RNA ENST00462717 [26] inhibit glioma cell proliferation by targeting MDM2, which are in agreement with our findings and collectively indicate that MDM2 plays a vital role in maintaining or even promoting glioma cell proliferation. It is likely that MDM2-regulated cell cycle progression is associated with its function in glioma cell proliferation [39, 40]. Moreover, MDM2 is also involved in the regulation of apoptosis and chemoresistance of glioma cells [41, 42]. Therefore, in addition to its functional role in inhibiting glioma cell proliferation, whether miR-585 affects other biological processes of glioma cells, such as migration, invasion, apoptosis, and chemoresistance, merits more research efforts in the future.

## Conclusion

we identify miR-585 as a novel dysregulated miRNA in gliomas and also an anti-proliferative regulator in glioma cells, wherein the targeted MDM2 plays a critical role. However, the molecular mechanisms of miR-585 function in glioma cell proliferation are not fully unveiled, suggesting that other unknown targets of miR-585 may exist to mediate its anti-proliferative function. Addressing this issue could help us to better exploit miR-585 as a therapeutic target in glioma treatment.

## Supplementary information

**Additional file 1.** mir-585 expression and its association with clinicopathologic features of glioma patients.

**Additional file 2.** Quantification data of Figure 5C and 5F.

## Data Availability

All data generated or analyzed during this study are included in this published article.

## Abbreviations

MDM2: Murine double minute 2
NSCLC: Non-small-cell lung cancer
ATCC: American Type Culture Collection
DMEM: Dulbecco’s modified Eagle’s medium
FBS: Fetal bovine serum
HE: Hematoxylineosin
GFP: Green fluorescent protein
OSCC: Oral squamous cell carcinoma
