# Supplementary figures and images for: MicroRNA-585 inhibits human glioma cell proliferation by directly targeting MDM2
Source: Cancer Cell Int. 2020 Sep 29;20:469. doi: 10.1186/s12935-020-01528-w (PMC7523344; doi:10.1186/s12935-020-01528-w)

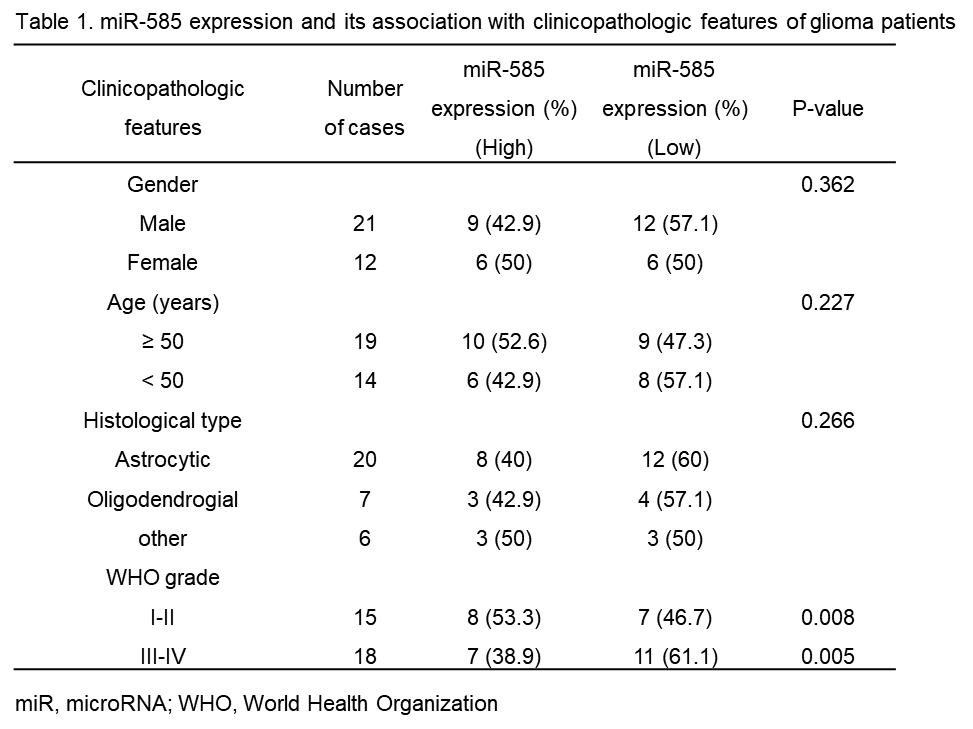

Supplement: Supplementary file 1 — Additional file 1. mir-585 expression and its association with clinicopathologic features of glioma patients. [file 12935_2020_1528_MOESM1_ESM.doc]

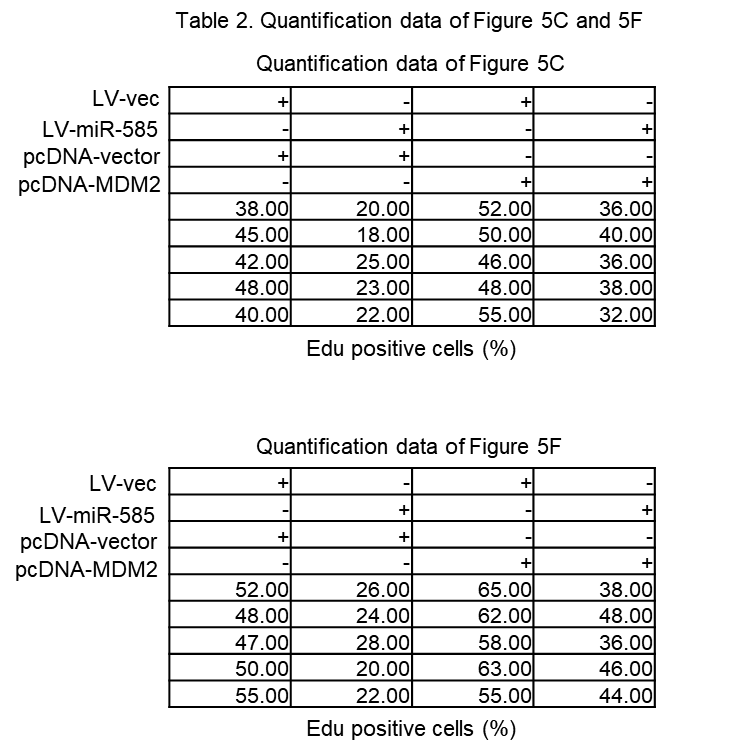

Supplement: Supplementary file 2 — Additional file 2. Quantification data of Figure 5C and 5F. [file 12935_2020_1528_MOESM2_ESM.doc]
